# An atlas of the bone marrow bone proteome in patients with dysproteinemias

**DOI:** 10.1038/s41408-023-00840-8

**Published:** 2023-04-28

**Authors:** Matthew Ho, Surendra Dasari, Alissa Visram, Matthew T. Drake, M. Cristine Charlesworth, Kenneth L. Johnson, Ganesh P. Pujari, Dragan Jevremovic, Taxiarchis Kourelis

**Affiliations:** 1 Division of Hematology, Department of Medicine, Mayo Clinic, Rochester, MN USA; 2 Division of Biomedical Statistics and Informatics, Department of Quantitative Health Sciences, Mayo Clinic, Rochester, MN USA; 3 Ottawa Hospital Research Institute, Ottawa, ON Canada; 4 Division of Endocrinology, Department of Medicine, Rochester, USA; 5 Proteomics Core, Mayo Clinic, Rochester, MN USA; 6 Department of Laboratory Medicine, Division of Hematopathology, Rochester, USA

**Keywords:** Cancer microenvironment, Myeloma

## Abstract

Multiple myeloma (MM) bone disease is a significant cause of morbidity but there is a paucity of data on the impact of malignant plasma cells on adjacent trabecular bone within the BM. Here, we characterize the proteome of trabecular bone tissue from BM biopsies of 56 patients with monoclonal gammopathy of undetermined significance (MGUS), smoldering (SMM), newly diagnosed (NDMM), relapsed MM (RMM), and normal controls. Proteins involved in extracellular matrix (ECM) formation and immunity pathways were decreased in SMM and active MM. Among the proteins most decreased were immunoglobulins, type IV collagen, and TIMP3, suggesting increased immunoparesis and decreased ECM remodelling within trabecular bone. Proteins most increased in SMM/MM were APP (enhances osteoclast activity), ENPP1 (enhances bone mineralization), and MZB1 (required for normal plasmablast differentiation). Pathway analyses showed that proteins involved in gamma -carboxylation, a pathway implicated in osteocalcin function, osteoblast differentiation, and normal hematopoiesis, were also overexpressed in SMM/MM. This study is the first comprehensive proteomic atlas of the BM bone proteome in dysproteinemias. We identify new key proteins and pathways for MM bone disease and potentially impaired hematopoiesis, and show for the first time that gamma -carboxylation pathways are increased in the bone tissue of SMM/MM.

## Introduction

Multiple myeloma (MM) is an incurable cancer characterized by clonal proliferation of long-lived plasma cells within the bone marrow (BM). Prior to the development of extramedullary disease, MM cells are dependent on the BM microenvironment for survival and progression from monoclonal gammopathy of undetermined significance (MGUS) and smoldering MM (SMM) to MM [1, 2]. The BM microenvironment is a complex interconnected network of cellular (e.g., mesenchymal stem or stromal cells (MSCs), osteoclasts/osteoblasts/osteocytes, endothelial cells, immune cells) and noncellular (e.g., extra-cellular matrix (ECM) proteins, cytokines, exosomes, growth factors) components [1]. The tumor milieu is not a static bystander and alterations occur as early as the MGUS stage [2]. Despite being a significant cause of MM-related morbidity, with up to 70% of patients presenting with myeloma bone disease at diagnosis [3], there is a paucity of studies investigating the role that trabecular bone plays in MM pathogenesis.

Congruent with Stephen Paget’s “seed and soil” hypothesis which maintains that cancer cells (seeds) will only metastasize to sites where the local microenvironment is favorable (i.e., the premetastatic niche), MM cells transform the local BM niche by dysregulating the release of soluble factors and exosomes, remodeling the BM ECM proteome, and altering the composition, function, and interactions of surrounding cellular compartments [4, 5]. It is well known that MM cells disrupt bone homeostasis by inhibiting osteoblast production, maturation, and activation in favor of osteoclastogenesis, ultimately leading to bone destruction [2]. However, a more in-depth analysis of the unique bone tissue proteome of myeloma is lacking owing to challenges in bone tissue enrichment, demineralization of its calcified matrix, and detection of low-abundance proteins.

Trabecular bone is composed of a highly calcified bone matrix and cellular components comprising osteocytes, osteoblasts, and osteoclasts (osteocytes make up 90–95% of cells embedded in the mineralized bone matrix) [6]. The bone matrix is roughly comprised of 40% ECM and related proteins (of which 90% is type I collagen and 10% noncollagenous proteins) and 60% calcium hydroxyapatite [7]. Bone is a highly dynamic and metabolically active tissue that undergoes constant remodeling [8]. Proteomics has recently emerged as a powerful tool to study bone metabolism and disease [8–10]. However, this method has not been applied to cancer-related bone disease, and specifically to bone disease in MM which is a prototypic model of a cancer that grows within the BM and can cause extensive bone damage.

In this study, we characterize the proteome of trabecular bone tissue in BM biopsies from patients with MGUS, SMM, newly diagnosed and relapsed MM, as well as localized amyloidosis (AL) controls. Our findings provide the first comprehensive proteomic atlas of the BM bone proteome in dysproteinemias. We identify new key proteins implicated in MM bone disease and show, for the first time, that proteins involved in gamma-carboxylation pathways are overexpressed in the trabecular bone in patients with SMM/MM compared to normal controls and MGUS.

## Materials and methods

We included 68 patients with MGUS (*n* = 12), smoldering MM (SMM; *n* = 11), MM within 6 months of diagnosis (*n* = 15) and relapsed MM (RMM; *n* = 18) as well as localized AL amyloidosis control samples (AL; *n* = 12). For patients with MGUS and SMM, all samples were collected at time of diagnosis. For patients with MM, samples were collected within 6 months of diagnosis or at time of relapse. All patients had consented to have their BM samples and clinical data used for research purposes, and this study was approved by the Mayo Clinic Institutional Review Board. The electronic medical records were reviewed to obtain clinical characteristics and treatment information for included subjects. Control samples were from patients with localized amyloidosis without evidence of a clonal plasma cell or B cell population in their BM or a circulating monoclonal protein in their serum or urine. Hematologic response and progression/relapse were defined according to IMWG criteria [11].Triple refractory patients were defined as patients refractory to a proteasome inhibitor, an immunomodulating agent and an anti-CD38 monoclonal antibody.

All but one of the RMM and MM within 6 months of diagnosis (total of 32 patients) also had paired cytometry by time of flight (CyTOF) data from BM immune cells, transcriptomic data from BM malignant cells and Luminex data for 65 proteins from BM plasma obtained from the same BM sample as the bone proteome. Details on CyTOF, Luminex and transcriptomic analyses of these samples have been previously published [12].

### Processing of samples and label-free MS

Eight-millimeter sections from demineralized formalin fixed paraffin embedded BM biopsy tissues were collected on polyethylene naphthalate membrane slides (ThermoFisher Scientific). The slides were deparaffinized and rehydrated before sample collection. Areas of trabecular bone were selected, cut, and captured by laser pressure catapulting (Zeiss MicroBeam) into the cap of a 0.5 mL tube containing 35 μL of digest buffer (100 mM Tris, pH 8.2/0.005% Zwittergent Z3-16). Approximately 2 cm^2^ area of tissue was collected per sample. Tubes were inverted and spun at 14 000 × *g* for 2 min to pellet the buffer and tissue. Samples were heated at 98 °C for 1 h with shaking at 800 rpm in a ThermoMixer C equipped with an insulating ThermoTop to ensure even heating and no evaporation of the buffer. Proteins were reduced and alkylated with 5 mM Tris (2-carboxyethyl) phosphine hydrochloride (Sigma) and 5 mM iodoacetamide, respectively. Sample volumes were increased to 100 μL, and samples subjected to ultrasonication using the BioRuptor Pico (Diagenode) for 5 cycles (30′ on, 30 s off) to further extract protein from the tissue. 0.5 μg of Trypsin (Promega) was added to each sample tube and incubated at 37 °C for 18 h with shaking at 800 rpm. Samples were acidified with trifluoroacetic acid and desalted on C18 cartridges using the Bravo automated liquid handling platform (Agilent). Final peptide concentrations were determined by fluorescent peptide assay (ThermoFisher) and 1.5 μg of each sample was aliquoted for nano liquid chromatography-tandem mass spectrometry (LC-MS/MS).

LC-MS/MS data was acquired on an Orbitrap Exploris480 mass spectrometer (Thermo Fisher, Bremen Germany), interfaced with a Dionex 3000 RSLC liquid chromatograph. Peptides were separated on a 100 µm i.d. by 40 cm long fused silica column self-packed with Acclaim RSLC 2.2 µm, 120 Å C18 stationary phase using an exponential gradient (curve 6) of 2%B mobile phase to 35% B at 80 min, followed by an exponential gradient from 35 to 45% B over 5 min, followed by a 3 minute ramp to 85% B, held for 3 min, then re-equilibrated at 2% B. Mobile phase A was 2% acetonitrile in water with 0.2% formic acid, mobile phase B was 80% acetonitrile, 10% isopropanol, 10% water with overall 0.2% formic acid. Column flow rate was 400 ƞL/min. Samples were loaded via autosampler and pre-concentrated on a 0.33 µL EXP2 stem trap packed with Halo 2.7 µm Peptide ES-C18 (Optimize Technologies, Oregon City, Oregon) for 5 min at 10 µL/min before switching the trap in-line with the separation column during the gradient.

Mass spectrometry data were collected using data dependent acquisition of tandem mass spectra (MS2) from peptide precursor masses (MS1) using a 2 second cycle. MS1 data were collected from m/z 340–1800 Thompson (Th) using resolving power of 60,000 (fwhm at m/z 200), normalized automatic gain control (AGC) of 300%, with a maximum ionization time (maxIT) of 100 ms. MS2 spectra were collected from a precursor range of 340–1400 Th, charge range (z) = 2–5, at 30000 resolving power, using a precursor isolation window of 1.2 Th, maxIT = 70 ms, normalized collision energy of 30%, minimum precursor intensity of 7E4, normalized AGC = 70%, and MS2 first mass = 120 Th. Precursor masses selected for MS2 spectra were subsequently excluded for 30 s.

Representative pictures of bone tissue before and after laser microdissection demonstrate the accuracy of this method and are shown in supplementary Fig. 1.

### Bioinformatics and statistical analysis

A previously published bioinformatics pipeline was utilized to process the raw LC–MS/MS data and perform peptide intensity-based label-free quantification of proteins present in the samples [13]. Raw data files were loaded into MaxQuant software (version 1.6.0.16) configured to search the MS/MS spectra against a database containing Uniprot human protein sequences and common contaminants [14]. Reversed protein sequences were appended to the database to estimate peptide and protein false discovery rates (FDRs). MaxQuant was instructed to use trypsin as digestion enzyme and the following posttranslational modifications when matching the MS/MS against the sequence database: carbamidomethyl cysteine (+57.021 Da), oxidation of methionine (+15.995), and deamidation of asparagine (+0.985). The software identified the peptides and proteins present in the samples at an FDR ≤ 1%, grouped protein identifications into groups and reported protein group intensities. Spectral counts, normalized to the total spectral counts in each sample, were used as a semi-quantitative measure of abundance.

A previously published, in-house developed R script was utilized to process the reported protein group intensities and identify differentially expressed proteins between any two experimental groups [13]. For this, protein group intensities of each sample were log2 transformed and normalized using trimmed mean of M-values (TMM) method [15]. For each protein group, the normalized intensities observed in any two experimental groups of samples were modeled using a Gaussian-linked generalized linear model. An ANOVA test was utilized to detect the differentially expressed protein groups between pairs of experimental groups. Differential expression p-values were FDR corrected using Benjamini–Hochberg–Yekutieli procedure. Protein groups with an FDR < 0.05 and an absolute log2 fold change of at least 0.5 were considered significantly differentially expressed and were saved for further analysis.

JMP 14.1 was used for statistical analyses (SAS Institute, Cary, NC). The Kruskal–Wallis statistical test was used to describe differences between groups. Kaplan–Meier survival analysis was used to estimate the overall survival (defined as time between sample collection and death or last follow-up) and progression-free survival (defined as time between sample collection and death/disease progression or last follow-up). Pearson’s correlation was used to test correlations between continuous variables. Correlation analyses were performed using the cluster variables function of JMP. This algorithm creates groups of the most highly correlated proteins and ranks groups according to how much of the variability of the dataset each group explains. Dimensionality reduction was performed using Uniform Manifold Approximation and Projection (UMAP) in the omiq.ai online platform [16].

### Pathway analyses

Pathway analyses were performed using WebGestalt [17], using only the differentially expressed proteins between the groups of interest and using the Reactome (pathway) as the functional database and an FDR corrected p-value of <0.05 when identifying significantly over-represented pathways. The protein-coding genome was used as the reference set and weighted set cover as a redundancy reduction method. In addition, gene set enrichment analyses (GSEA) [18] using the Reactome database, was performed to identify differentially expressed (FDR < 0.05) gene pathways between groups and as a confirmatory method. A comprehensive list of proteins within each pathway will not be provided but can be easily reproduced using available raw data (see supplemental file: raw protein MS1 count.xlsx).

## Results

### Patient characteristics

We included a total of 56 patients (12 MGUS, 11 SMM, 15 MM within 6 months of diagnosis, 18 RMM) and 12 normal controls. Their baseline characteristics are shown in the Table 1. There was no significant age difference between the median age of cases compared to that of controls (66 years vs 61 years, respectively, *p* = 0.18). High risk patients were overrepresented in this study (53%), which was reflected in the short time to progression for newly diagnosed and relapsed patients (median of 15 and 5 months, respectively).

**Table 1:** Baseline characteristics of patients.

	Median (range) or *N* (%)
Total number of patients	68 (100%)
Diagnosis type	
Localized AL controls	12 (18%)
MGUS	12 (18%)
Smoldering MM	11 (16%)
MM within 6 months from diagnosis^a^	15 (22%)
Relapsed MM^b^	18 (26%)
Age at sample collection, years	65 (43–85)
Female sex	31 (46%)
High risk FISH^c^	20 (53%)
Follow-up from diagnosis, months (controls excluded)	57 (3–154)
BMPC % at sample collection	
MGUS	2.5% (0–7.5%)
Smoldering MM	14% (8–50%)
Active MM	50% (0–90%)
Time to hematologic progression from sample collection, months	
MM within 6 months from diagnosis	15 (5–68)
Relapsed MM	5 (1–33)
Received osteoclast inhibitors within 6 months^d^ (controls excluded)	20/56 (36%)
MGUS	1/12 (8%)
SMM	2/11 (18%)
Active MM	17/33 (52%)
Any skeletal fracture within 6 months from sample collection^e^	15 (45%)

FISH fluorescent in-situ hybridization, MM multiple myeloma.

^a^Of which 12 were newly diagnosed and 3 patients were after induction therapy (1 partial response and 2 very good partial responses).

^b^Of which 12 triple-refractory.

^c^Considered for smoldering and active MM and only for the 38 patients with complete FISH data. Defined as presence of deletion 17p, t (4;14), +1q, t(14;16) or t(14;20).

^d^Of which 14 patients received zoledronic acid, 3 alendronate and 3 pamidronate.

^e^Considered for the 33 patients with active MM.

### The BM bone proteome is a diverse ecosystem of proteins that varies significantly across patients

**Fig. 1:**
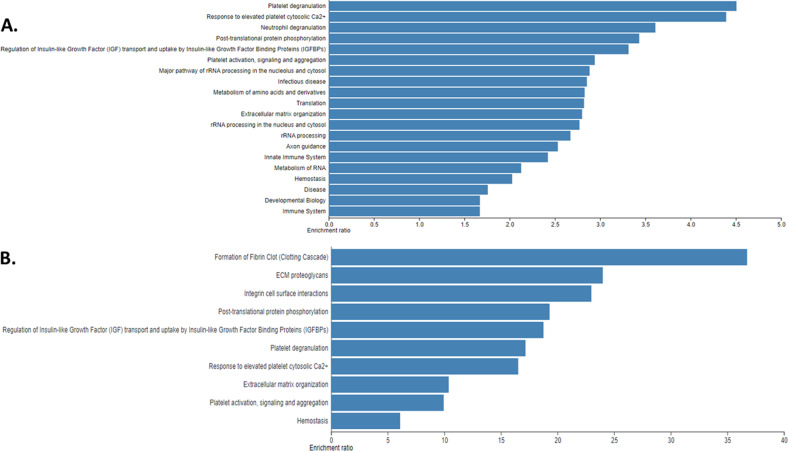
Overview of the diversity of the bone marrow bone proteome. **A** WebGestalt analysis of all proteins identified across all samples using the Reactome pathway and presenting the top 20 pathways. **B** WebGestalt analysis of the top 5% most abundant proteins. (top 10 pathways).

We identified a total of 1951 distinct proteins across all samples (raw protein MS1 counts.xlsx, supplementary material). There was no difference in the total proteomic content (total number of normalized spectral counts for all proteins in a sample) according to diagnosis type (data not shown). WebGestalt analyses of the top pathways associated with the entire proteome and the top 5% most abundant proteins are shown in Fig. 1. Proteins involved in structural support (“ECM organization” pathway) were, not surprisingly, overrepresented. Notably, proteins involved in protein translation machinery and immunity (the majority of which were immunoglobulins) were also overrepresented, suggesting that the bone tissue is a metabolically and immunologically active tissue. To visualize major patterns in the data across patients we performed a UMAP analysis of all patients using their entire proteome and normalized spectral counts as semiquantitative measures of abundance. No significant grouping of patients was apparent (supplemental Fig. 2), suggesting significant variability in the BM bone proteome even within the same diagnostic categories (e.g., MGUS or SMM or MM). To identify which proteins were responsible for most of the variability across patients we performed PCA. The first 2 principal components explained 15.1% and 6.4% of the variability in the data, respectively. We then performed WebGestalt pathway analyses in the top 5% of proteins with the highest and lowest loadings, respectively for each principal component (supplemental Figs. 3, 4). This demonstrated that proteins with high loadings within principal component 1, were mostly proteins involved protein translation whereas those with low loadings were proteins involved in the activation of the complement cascade, keratinization, and ECM formation. Similarly, proteins with high loadings within principal component 2, were involved in ECM formation and those with low loadings were involved in protein translation.

### Normal controls and MGUS patients have a distinct proteome compared to those with higher plasma cell burden states

**Fig. 2:**
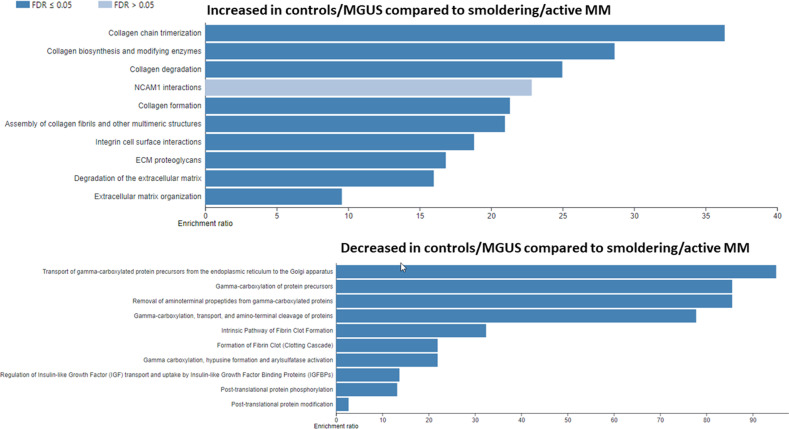
Proteomic differences between patients with low and high plasma cell burden. A WebGestalt analysis of all proteins differentially expressed between controls/MGUS and smoldering active multiple myeloma using the Reactome pathway and presenting the top 10 pathways. MM multiple myelom.

**Fig. 3:**
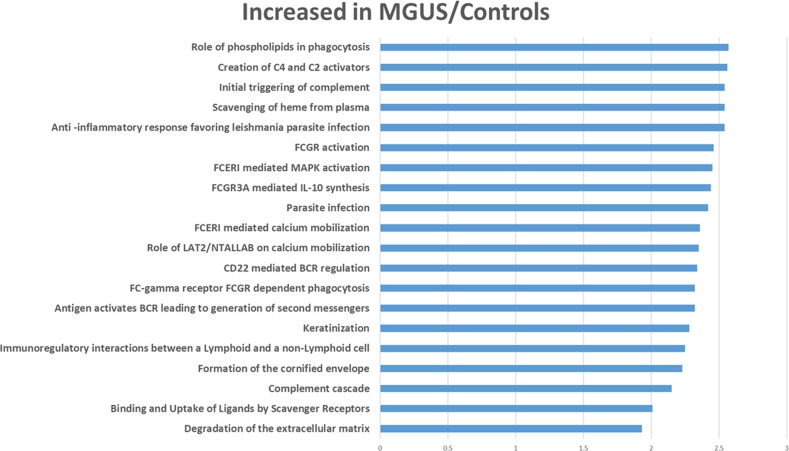
Gene set enrichment analyses of proteins in MGUS and control samples compared to smoldering and active myeloma samples (only the top 20 pathways are reported). The values on the X axis are normalized enrichment scores and all were significant at an FDR corrected *p* value of <0.05.

**Fig. 4:**
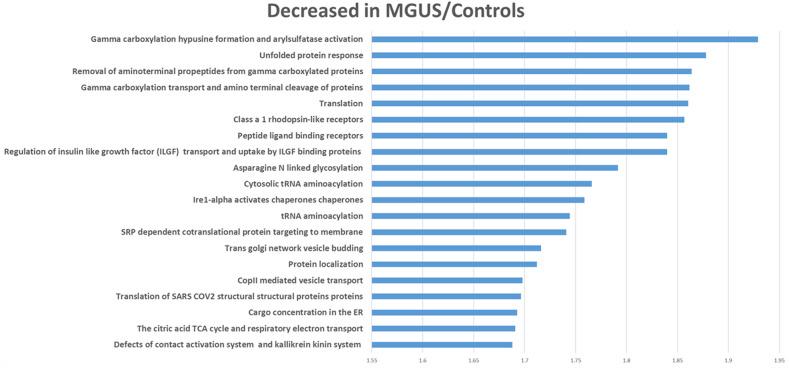
Gene set enrichment analyses of proteins in smoldering and active myeloma samples compared to MGUS and control samples (only the top 20 pathways are reported). The values on the X axis are normalized enrichment scores and all were significant at an FDR corrected *p* value of <0.05.

Given the high level of variability observed and the limited number of cases within each group, we combined normal controls and MGUS cases together (i.e., low plasma cell burden) and compared them to SMM and MM cases (i.e., high plasma cell burden) to increase statistical power. The results of a WebGestalt analysis are shown in Fig. 2. The abundance of proteins involved in ECM formation pathways were decreased whereas proteins involved in gammacarboxylation pathways were increased in SMM/MM. Amongst the proteins (supplemental material) most decreased in SMM/MM were immunoglobulin genes, collagen 4 and tissue inhibitor of metalloproteinase 3 (TIMP3). Among the proteins most increased in SMM/active MM were amyloid precursor protein (APP), ectonucleotide pyrophosphatase/phosphodiesterase 1 (ENPP1), Lectin, Mannose Binding 2 (LMAN2), Marginal Zone B And B1 Cell Specific Protein (MZB1) and X-Prolyl Aminopeptidase 1 (XPNPEP1). A GSEA analysis largely confirmed the above findings (Figs. 3 and 4, full list of proteins within each pathway is provided as supplemental data).

Within the most upregulated pathways in the MGUS/control groups were immunoglobulin proteins (dominating all pathways except those relating to keratinization or ECM formation). Protein pathways upregulated in SMM/MM cases again included those involved in gamma-carboxylation, endoplasmic reticulum (ER) stress response, and protein trafficking within cells. All differentially expressed proteins and a ranked list used for WebGestalt and GSEA analyses, respectively, are included as supplemental data. No significant pathway differences were identified between patients with newly diagnosed MM and relapsed or smoldering MM. We also compared the following groups: males versus females, patients receiving osteoclast inhibitors (bisphosphonates or denosumab) versus not, high risk versus non-high risk active MM patients (per IMWG criteria) and patients with a bone fracture within 6 months from sample collection but found no differences.

We have previously published CyTOF data from immune cells, Luminex data from BM plasma proteins, and transcriptomic data from malignant plasma cells collected from the same 15 patients with MM diagnosed within 6 months and all but one RMM patient (*n* = 17) [12]. We hypothesized that bone proteins having a high correlation with any of these other BM components (immune cells, BM plasma proteins or malignant cell genes) would be more likely to coregulate or influence each other. We interrogated a correlation matrix of bone proteomic data with the previously reported CyTOF, Luminex and transcriptomic data from BM immune cells, plasma proteins and malignant plasma cells respectively, and, using a cutoff of Pearson’s R > 0.9 (R 2 > 0.8), however we identified no significant correlations.

## Discussion

This study represents the first comprehensive proteomic atlas of the bone marrow bone proteome in various dysproteinemias. We show that the bone is a metabolically and immunologically active tissue. We demonstrate that bone from patients with active or smoldering MM has lower levels of ECM related proteins compared to those from controls and MGUS, suggesting that the loss of ECM proteins is associated with decreased structural rigidity found in more advanced dysproteinemias. We also show that gamma-carboxylation pathways are activated in SMM and active MM, and identify several novel proteins involved in bone physiology that are differentially expressed between the two groups.

Metalloproteinases are collagenases that promote collagen breakdown and bone destruction. MM cells have the ability to induce expression of metalloproteinases in the surrounding BM stromal cells [19]. Interestingly, we identified that a collagenase-inhibitor, TIMP3, was amongst the most abundant proteins in control and MGUS patients suggesting that TIMP3 may be downregulated in active MM. Of note, TIMP-3 is unique among TIMP family members in that it becomes tightly bound to the ECM shortly after secretion [20, 21], which may explain why overall TIMP3 expression was higher in AL/MGUS, which also express much higher levels of normal ECM proteins, as compared to SMM/MM. Functionally, TIMP3 regulates ECM remodeling and deficient mice have decreased overall bone integrity [22]. TIMP3 is associated with lower levels of inflammatory cytokines in normal tissue [23] and in MM, since it can inhibit soluble IL-6 receptor production by MM cells [24]. This suggests that TIMP3 loss may be permissive for the development of pro-inflammatory microenvironment that is associated with MM development.

Interestingly, APP, the most differentially abundant protein in bone tissues obtained from patients with active or smoldering MM in our study, is also elevated in osteoporotic bone tissues where it enhances osteoclast function [25]. This is the first report to identify increased APP in MM or SMM and further studies are needed to clarify the functional role of APP in this setting. ENPP1, another differentially expressed protein in bone tissues of SMM/MM, is also preferentially upregulated in BM long-lived plasma cells (the nonmalignant counterpart to MM cells) [26]. Among its many functions, ENPP1 has also been shown to serve as an adhesion molecule associated with glycosaminoglycans in the ECM and is also expressed by plasma cells [26, 27]. Importantly, ENPP1 homozygous knockout mice produced significantly reduced number of BM long-lived plasma cells following immunization, showing that ENPP1 plays an important role in long-term plasma cell survival [26]. Finally, MZB1, is required for normal plasmablast differentiation [28]. These observations collectively suggest that the bone tissue in MM may actively support MM growth via MZB1 and ENPP1.

We were intrigued to identify an increase in proteins involved in gamma-carboxylation in both smoldering and active MM. Indeed, gamma-carboxylation is implicated in the vitamin K-stimulated function of osteocalcin, a major noncollagenous protein of the bone matrix that contains three gamma-caboxyglutamic acid residues [29]. Furthermore, warfarin, a well-known inhibitor of gamma-carboxylation of clotting factors, can also inhibit osteoblastic differentiation [30] and has been shown to inhibit normal hematopoiesis in the BM partly via the decarboxylation of periostin [31]. Furthermore, only 2 patients with SMM/MM and 3 patients with MGUS/Localized AL received warfarin, which suggests that this would not have biased our results. Our findings may therefore be a result of homeostatic mechanisms activated within the bone tissue as a result of MM-induced bone loss and myelosuppression. However, it appears that the beneficial effects of gamma-carboxylated pathways on bone are countered by other mechanisms of MM-induced bone loss.

Our study has several limitations. High risk patients were overrepresented in our study which was reflected in their short remission lengths. This likely reflects a selection bias, i.e. more likely to come to a referral institution and to have research samples available but limits the generalizability of our results. In addition, we noticed significant variability across groups so our study was not powered to detect differences across various subgroups but can help with the design of future studies. Finally, even though we took care to microdissect bone and avoid surrounding tissue, the degree of “contamination” by BM plasma or surrounding tissues and cells is unclear. Therefore, we were unable to ascertain to what degree these proteins were exclusive to the bone tissue or not.

In summary, we demonstrate that bone physiologic changes can be detected and quantified in the marrow bone proteome using shotgun proteomics. We identify new key proteins and pathways for MM bone disease and potentially impaired hematopoiesis, and show for the first time that gamma-carboxylation pathways are increased in the trabecular bone tissue of patients with SMM/MM. Such proteomic signatures can be obtained from routine BM biopsy samples and have the potential to serve as biomarkers of MM bone disease if validated in larger studies. Data from this study will be made publicly available for use by the broader scientific community for hypothesis generation.

## Supplementary information

Supplemental Figure 1

Supplemental figure 2

Supplemental figure 3

Supplemental figure 4

spectral counts of all identified proteins

Reproducibility Checklist

## Data Availability

Protein spectral counts according to diagnosis groups have been submitted as supplemental material. Additional de-identified clinical information can be shared upon request.
