# Supplementary figures and images for: An atlas of the bone marrow bone proteome in patients with dysproteinemias
Source: Blood Cancer J. 2023 Apr 28;13(1):63. doi: 10.1038/s41408-023-00840-8 (PMC10140150; doi:10.1038/s41408-023-00840-8)

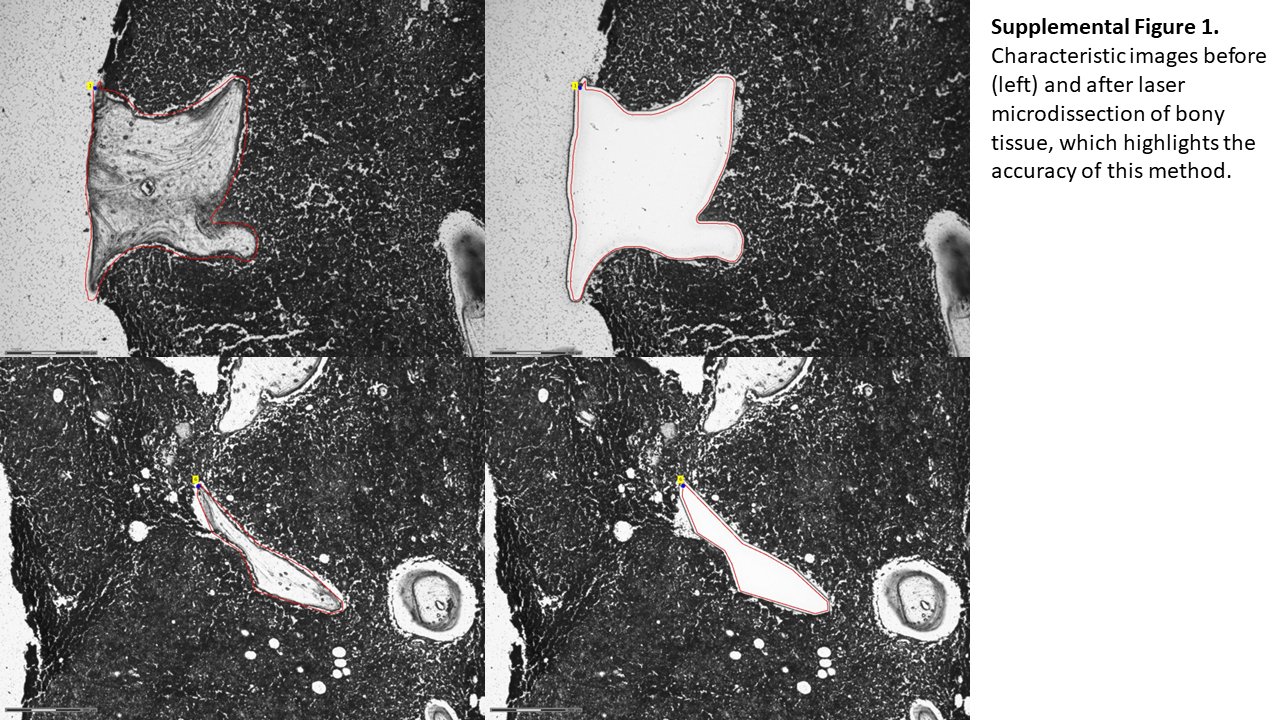

Supplement: Supplementary file 1 — Supplemental Figure 1 [file 41408_2023_840_MOESM1_ESM.tif]

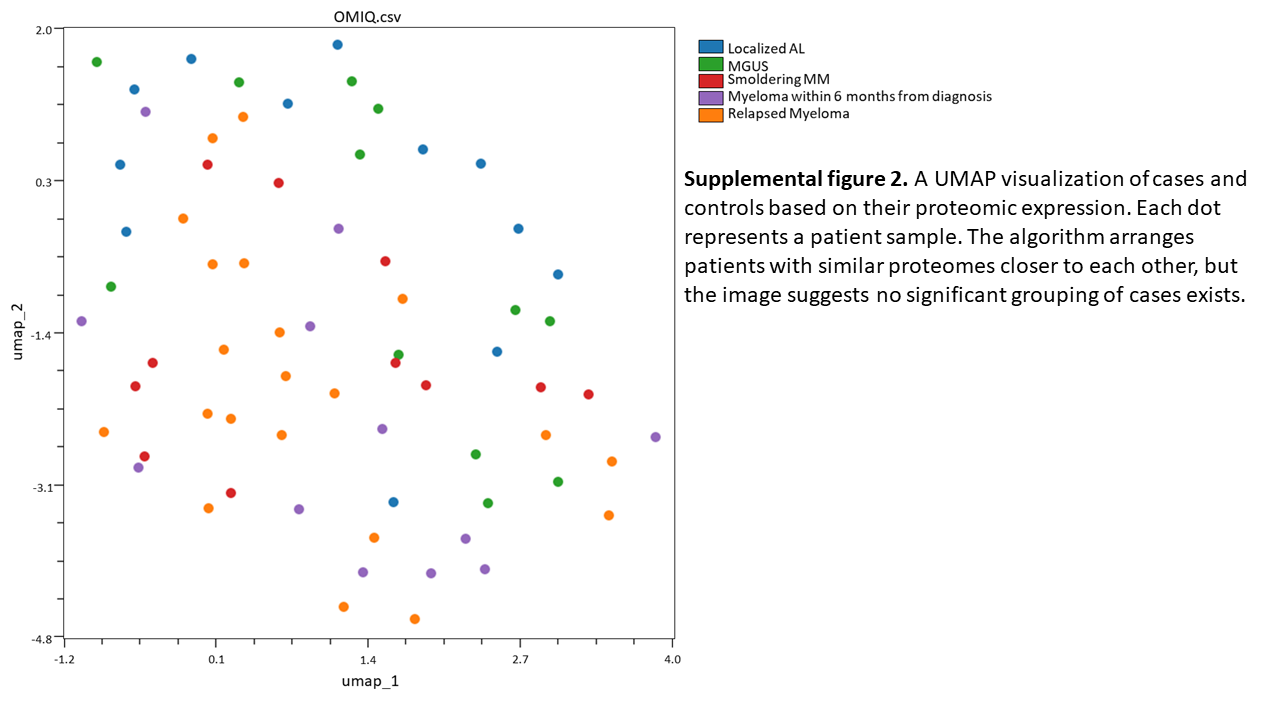

Supplement: Supplementary file 2 — Supplemental figure 2 [file 41408_2023_840_MOESM2_ESM.tif]

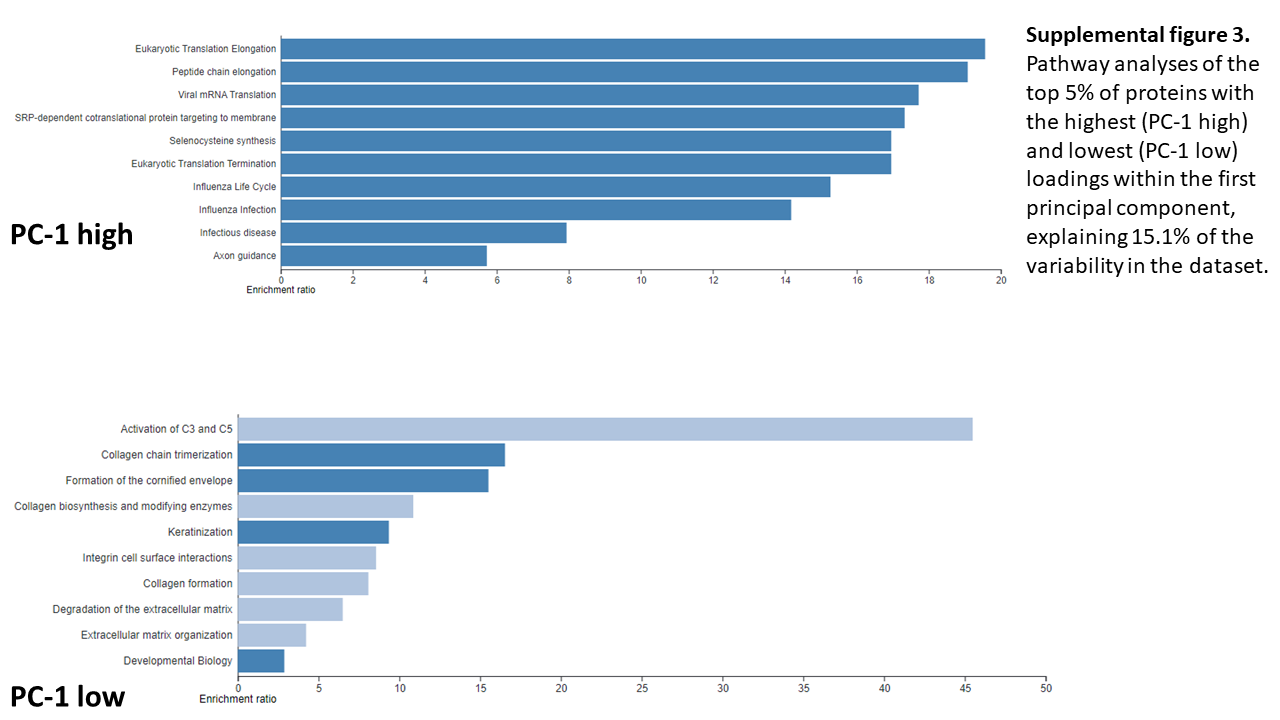

Supplement: Supplementary file 3 — Supplemental figure 3 [file 41408_2023_840_MOESM3_ESM.tif]

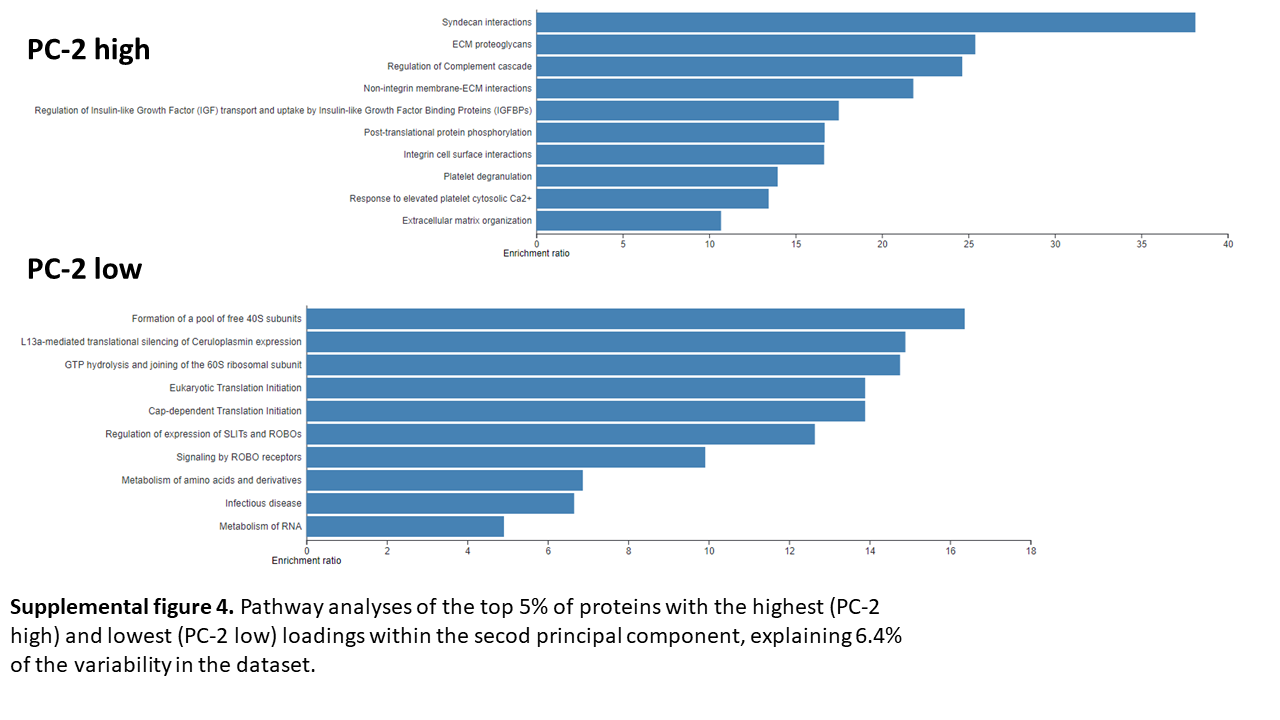

Supplement: Supplementary file 4 — Supplemental figure 4 [file 41408_2023_840_MOESM4_ESM.tif]
